# An Unusual Abscisic Acid and Gibberellic Acid Synergism Increases Somatic Embryogenesis, Facilitates Its Genetic Analysis and Improves Transformation in *Medicago truncatula*

**DOI:** 10.1371/journal.pone.0099908

**Published:** 2014-06-17

**Authors:** Kim E. Nolan, Youhong Song, Siyang Liao, Nasir A. Saeed, Xiyi Zhang, Ray J. Rose

**Affiliations:** School of Environmental and Life Sciences, The University of Newcastle, Callaghan, New South Wales, Australia; Virginia Tech, United States of America

## Abstract

Somatic embryogenesis (SE) can be readily induced in leaf explants of the Jemalong 2HA genotype of the model legume *Medicago truncatula* by auxin and cytokinin, but rarely in wild-type Jemalong. Gibberellic acid (GA), a hormone not included in the medium, appears to act in Arabidopsis as a repressor of the embryonic state such that low ABA (abscisic acid): GA ratios will inhibit SE. It was important to evaluate the GA effect in *M. truncatula* in order to formulate generic SE mechanisms, given the Arabidopsis information. It was surprising to find that low ABA:GA ratios in *M. truncatula* acted synergistically to stimulate SE. The unusual synergism between GA and ABA in inducing SE has utility in improving SE for regeneration and transformation in *M. truncatula.* Expression of genes previously shown to be important in *M. truncatula* SE was not increased. In investigating genes previously studied in GA investigations of Arabidopsis SE, there was increased expression of *GA2ox* and decreased expression of *PICKLE*, a negative regulator of SE in Arabidopsis. We suggest that in *M. truncatula* there are different ABA:GA ratios required for down-regulating the *PICKLE* gene, a repressor of the embryonic state. In *M. truncatula* it is a low ABA:GA ratio while in Arabidopsis it is a high ABA:GA ratio. In different species the expression of key genes is probably related to differences in how the hormone networks optimise their expression.

## Introduction

Somatic embryogenesis (SE) in addition to being useful as an *in vitro* system to study embryogenesis has facilitated the development of clonal propagation, somatic hybridisation and transformation for the study of genes and for transgenic crops. Auxin has been the central hormone since it was shown with carrot (*Daucus carota*) that auxin could induce SE and then the removal of auxin or lowering of the auxin concentration facilitated embryo maturation [1]. In the perennial *Medicago sativa*, callus initiated by an auxin plus cytokinin followed by a pulse of the synthetic auxin 2,4-D (2,4 dichlorophenoxyacetic acid) will induce SE [2]. The auxin NAA (1-naphthalene acetic acid) and the cytokinin BAP (6-benzylaminopurine) can induce somatic embryogenesis in suitable genotypes of the model legume *Medicago truncatula* [3], [4]. Cytokinin is essential in *M. truncatula* as with auxin alone roots are initiated [5]. In addition to the hormone component, the stresses induced during the preparation of the explant are an important component of SE [6], [7]. Indeed, stress alone is capable of inducing SE in some systems [8]. In this context the stress hormone abscisic acid (ABA) can induce SE in carrot root apices [9]. In *M. truncatula*, SE is enhanced by ABA when it is added to the auxin plus cytokinin required for SE induction [10]. This is not surprising given what is now known about how plant hormone signaling can influence gene expression [11].

Auxin and cytokinin are clearly central regulators in development *in vitro* and *in vivo.* What has been interesting in SE studies has been the demonstration that hormones not added to the medium but present in the explant’s tissue of origin or which are synthesised as a result of culture, influence the response of auxin and or cytokinin in regeneration. Ethylene is one example of a hormone which is not applied in the medium but is synthesised in culture, likely as a result of stress and auxin. Ethylene is required for auxin-induced SE in Arabidopsis [12] and auxin plus cytokinin-induced SE in *M. truncatula* [13]. In Arabidopsis [14] and carrot [15] gibberellic acid (GA) biosynthesis needs to be repressed as GA will act as a repressor of SE. An important early experiment in this context was the study by Ogas et al. [16] where the roots of the Arabidopsis *pickle* (*pkl)* mutant produced somatic embryos without hormones and this was repressed by GA.

In the major flowering plant model Arabidopsis, SE can be induced by auxin (synthetic auxin 2,4-D) alone in the medium [12], [17], [18] so this represents an important difference to *M. truncatula*. It is nevertheless informative to see the differences and commonalities with SE in the model legume *M. truncatula* to assist in providing a generic conceptual model of SE induction [19]. Given the importance of legumes in agriculture, it is also important to gain information that is perhaps specific to legumes, and which may increase the efficiency of transformation and regeneration in these often recalcitrant species. In *M. truncatula* ABA enhances SE [10] and ethylene inhibitors inhibit SE [13]. Arabidopsis shows similar responses as ABA mutants impair SE [20] and ethylene inhibitors inhibit SE [12]. Similarly a number of key genes are required for SE induction in both Arabidopsis and *M. truncatula*, for example *WUSCHEL* (*WUS*) [18], [21] *SERK1* [22], [23] and *SERF1* [12], [13]. There is however substantive work which indicates that endogenous GA needs to be down-regulated to facilitate SE [24]. Following the initial work with the *pkl* mutant in Arabidopsis [16] genes that induce or promote SE in Arabidopsis such as LEC transcription factors have been implicated in repressing GA activity [24], [25].

Given the importance of GA metabolism for SE in Arabidopsis and its inverse relationship with ABA [14], [15], [16], [24] it was important to investigate the GA response in *M. truncatula* to relate to our current understanding of the mechanism of SE in this legume model [19]. Unexpectedly, given the usual GA and ABA antagonism in physiological mechanisms [26] we found ABA and GA acted synergistically to enhance SE. We have taken advantage of this synergism to improve the transformation of *M. truncatula* and to probe its relationship to the expression of genes studied previously in *M. truncatula* and or Arabidopsis and implicated in SE. The gene expression studies indicate the subtleties involved in the timing and extent of gene expression and how networks may be modulated in different *in vitro* media and in different species. In Arabidopsis SE, *WUS* is induced by auxin [18] while *WUS* is induced by cytokinin in *M. truncatula* [21] and *PICKLE* (*PKL*) expression in Arabidopsis and in *M. truncatula* appears to require different ABA:GA ratios. In different species the same gene may be regulated by different hormones, so there may be considerable overlap of the genes required to be expressed for SE induction.

## Results

### The Effect of GA on Somatic Embryogenesis

**Figure 1:**
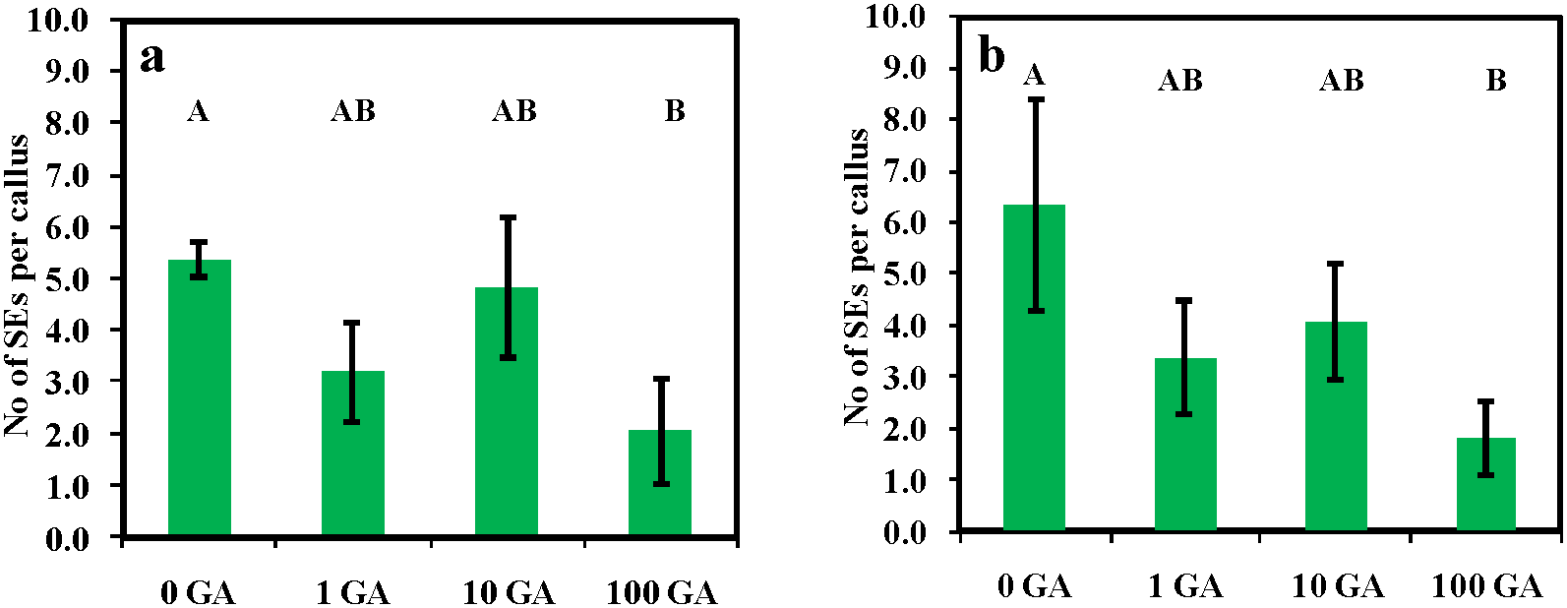
The effect of GA (0, 1, 10 and 100 µM) on somatic embryogenesis induced by P4 10∶4 (NAA:BAP in µM) for three weeks before transfer to P4 10∶4∶1 (NAA:BAP:ABA in µM) shown for two experiments (a) and (b). Five plates of each treatment with six explants per plate. Counts were made 11 weeks from the initiation of culture. Treatments with different letters are significantly different at the 0.05 probability level; vertical bars indicate ± standard error. The numbers on the X-axis represent the GA concentration in µM.

Our standard protocol for SE is incubation in auxin plus cytokinin for three weeks then subculture into auxin plus cytokinin plus ABA. GA when applied with auxin plus cytokinin from the beginning of leaf explant incubation had little effect on SE at common physiological concentrations of 1 µM and 10 µM (Fig. 1). However, at 100 µM GA was inhibitory. We then carried out further experiments to investigate the effect of GA when ABA was given at the beginning of explant incubation to see if this would give similar results.

### GA+ABA Enhances Somatic Embryogenesis Induced by Auxin+Cytokinin

**Figure 2:**
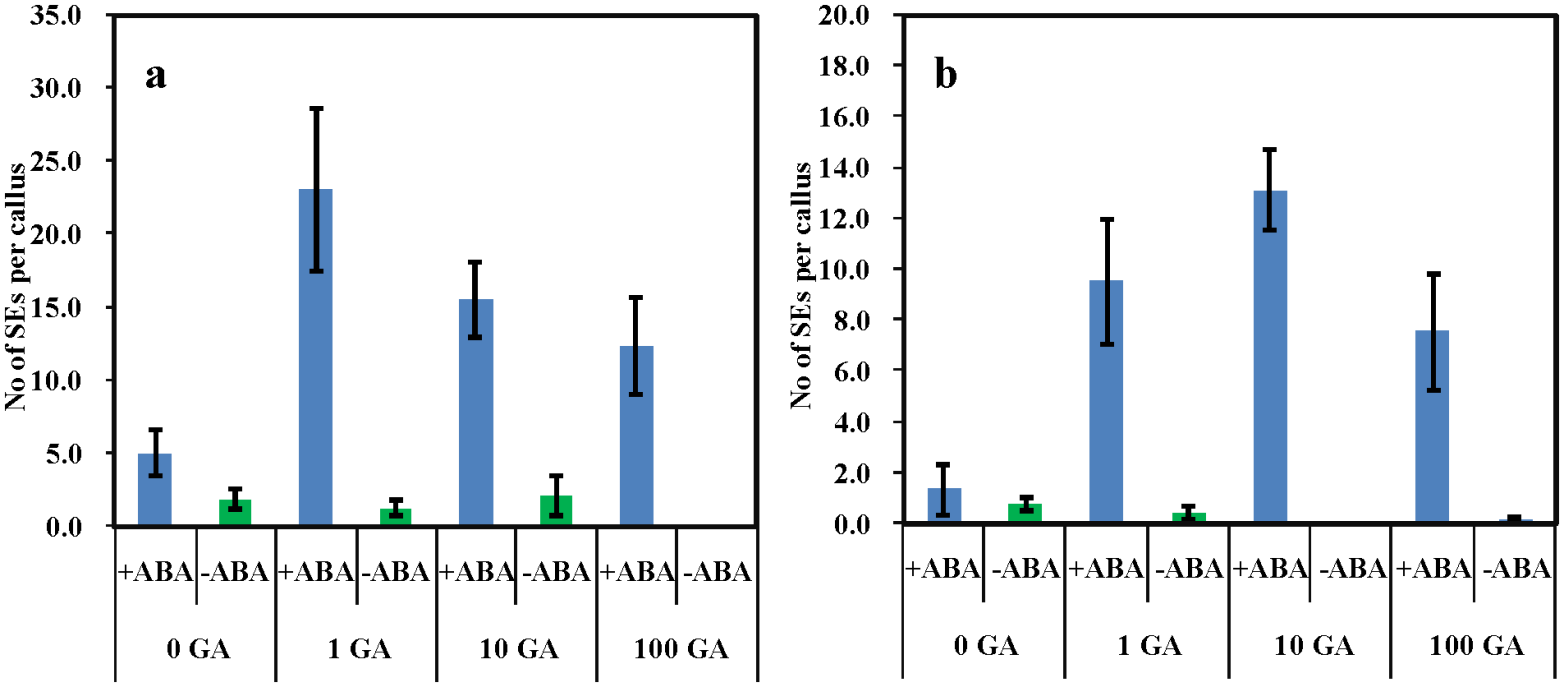
The effect of 1 µM ABA+GA at 0, 1, 10, or 100 µM) on somatic embryogenesis induced by P4 10∶4 (NAA:BAP in µM) shown for two separate experiments (a) and (b). Five plates of each treatment with six explants per plate. Counts were made 7 weeks after the initiation of culture. Vertical bars indicate ± standard error.

**Figure 3:**
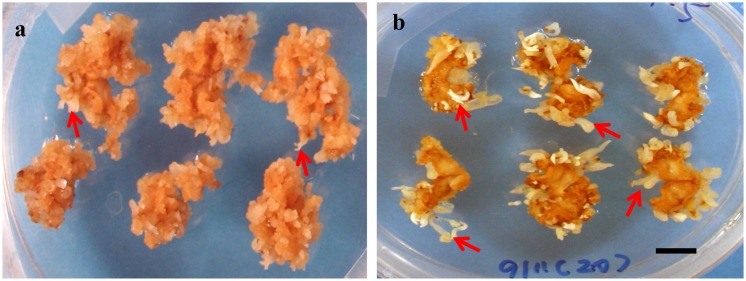
Somatic embryos from P4 10∶4 (NAA:BAP in µM) for 3 weeks then P4 10∶4∶1 (NAA:BAP:ABA in µM) (a) and a P4 10∶4∶1∶5 protocol (NAA:BAP:ABA:GA in µM) (b) 7 weeks after the initiation of culture. Arrows show somatic embryos. Bar = 1 cm.

What was unexpected was the clear enhancement of SE in the continued presence of 1 µM ABA plus different concentrations of GA (0,1,10, and 100 µM) in addition to the auxin (10 µM) plus cytokinin (4 µM), compared to the auxin (10 µM) and cytokinin (4 µM) alone (Fig. 2). This synergism between the usually antagonistic GA and ABA is most unusual. The morphology of the embryos (Fig. 3) is slightly different in that the hypocotyl is more elongated, characteristic of a GA effect on organ growth. Given that regeneration of *M. truncatula* could be clearly increased, further experiments were carried out using our transformation protocols [27], to examine if transformation efficiency of this legume model could be improved.

### Plant Transformation Test of P4 10∶4∶1∶5 Medium

**Figure 4:**
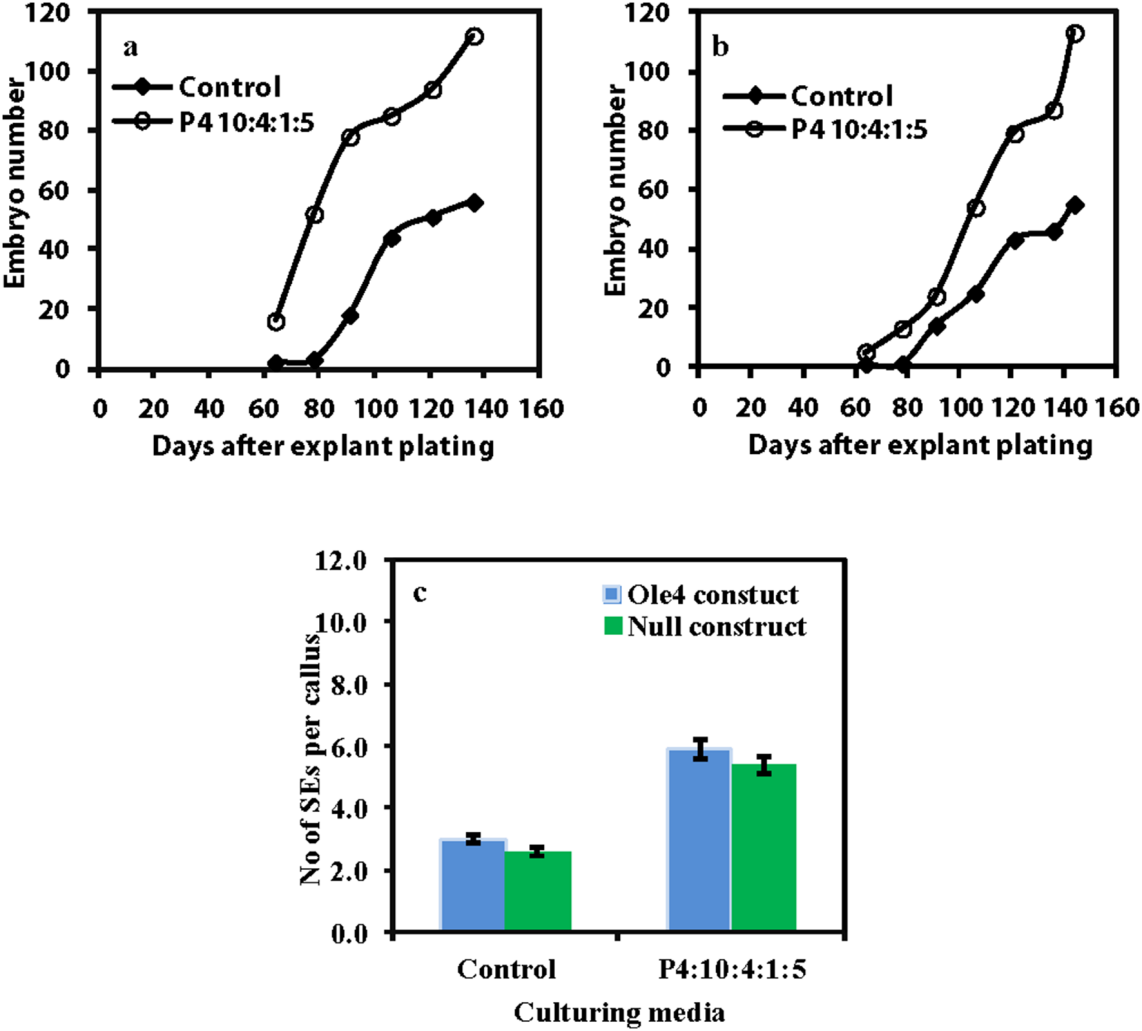
Time course of somatic embryo accumulation after culture initiation for transformation with the binary vector pMDC83 with a *MtOLEOSIN4* gene - MtOLEOSIN4 GFP (Ole4-GFP) (a) or with no inserted gene - Null-GFP (b), and total embryos produced per callus (c) (Vertical bars indicate 95% confidence interval).

Embryo accumulation after transformation using the standard P4 10∶4 (NAA:BAP in µM) for three weeks before transfer to P4 10∶4∶1 (NAA:BAP:ABA in µM), or P4 10∶4∶1∶5 medium (NAA:BAP:ABA:GA in µM) for two different constructs (see Table 1) is presented in Fig. 4. One construct had no inserted gene for transfer (null construct), while another construct had an *MtOLEOSIN4* gene [28] inserted. Hygromycin was in the media as the selection agent. Somatic embryos first appeared in GA+ABA treatments and a rapid increase in numbers occurred about 20 days earlier than the standard protocol (Fig. 4a, b). A large increase in total embryo numbers occurred in the treatments with ABA+GA with both the null construct and with the construct containing the *MtOLEOSIN4* gene (Fig. 4c). This showed that GA+ABA stimulated somatic embryogenesis under transformation conditions.

**Table 1:** The three phases of transformation with different media types used to culture tissue transformed with Ole4-GFP and Null-GFP constructs.

Incubation	Standard	ABA+GA
**Phase I**	P4 10∶4	P4 10∶4∶1∶5
**Phase II**	P4 10∶4, T+H	P4 10∶4∶1∶5, T+H
**Phase III**	P4 10∶4∶1, T+H	P4 10∶4∶1∶5, T+H

Note: ‘Standard’ refers to the control medium, P4 10∶4 (NAA:BAP in µM), P4 10∶4∶1 (NAA:BAP:ABA in µM); ‘ABA+GA’ refers to the ABA+GA treatment, P4 10∶4∶1∶5 (NAA:BAP:ABA:GA in µM); ‘T’ indicates timentin (750 µg mL^−1^) and ‘H’ indicates hygromycin (15 µg mL^−1^ Phase II and 20 µg mL^−1^ for Phase III).

### Expression of Selected Genes in Response to ABA+GA Stimulation of SE

Gene expression (transcript accumulation) was compared between the auxin plus cytokinin treatments and auxin plus cytokinin with ABA + GA. There were two reasons for gene selection. One group of genes has been studied in relation to genetic regulation of SE in *M. truncatula*, particularly in terms of hormones (*MtSERK1, MtWUS* and *MtSERF1*), and stress (*MtSK1* and *MtRBOHA*) influences. Another group of genes has been studied in Arabidopsis, particularly in relation to GA influences (*PKL*, *GA2ox*, *LEC1*) but have not been investigated in *M. truncatula*. *MtSERK1* is auxin-induced in *M. truncatula* [23], *MtWUS* is cytokinin-induced [21] and *MtSERF1* requires auxin, cytokinin and ethylene [13], [29]. The stress kinase *MtSK1* is up-regulated early in embryogenic cultures [7]. The NADPH oxidase (MtRBOH protein) is an important generator of ROS and in precursor studies we established *MtRBOHA* was expressed in the *M. truncatula* SE induction period. The *PKL, GA2ox*, and *LEC1* genes have been linked in Arabidopsis SE to a negative role for GA in SE [24].

**Figure 5:**
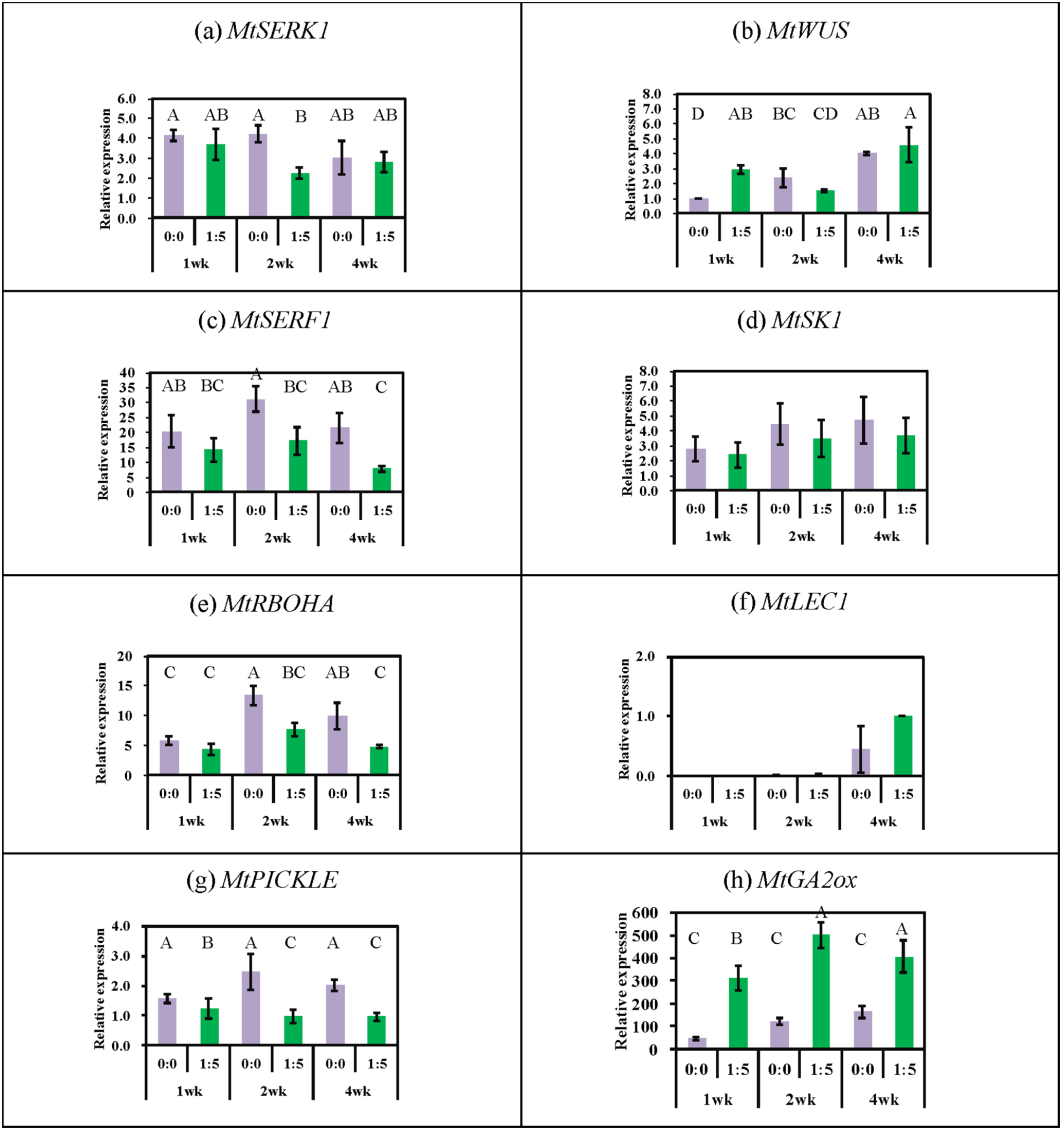
Comparisons of gene expression between P4 10∶4 (NAA:BAP in µM) and P4 10∶4∶1∶5 (NAA:BAP:ABA:GA in µM) treatments (shown as 0∶0 and 1∶5 respectively). Gene expression for cultured tissues at 1, 2 and 4 weeks was calibrated to expression in young leaf tissue (the explant source given the relative expression of 1) for all genes except *MtLEC1*, which is not expressed in leaf. *MtLEC1* expression was calibrated to expression at 4 weeks in P4 10∶4∶1∶5 medium. Treatments with different letters are significantly different at the 0.05 probability level; vertical bars indicate ± standard errors from three biological repeats.

At two weeks ABA+GA (Fig. 5a) caused a decrease in *MtSERK1* gene expression with no difference at weeks one and four. *MtWUS* was stimulated by ABA+GA at one week (Fig. 5b), but then expression showed no significant change. Expression of the *MtSERF1* transcription factor was reduced at 2 and 4 weeks by ABA+GA (Fig. 5c). The expression of *MtSK1* (Fig. 5d) was unchanged by ABA+GA while the expression of *MtRBOHA* (Fig. 5e) was reduced at weeks 2 and 4. The *M. truncatula* homologue of *LEC1* begins to be expressed at week 4 when embryos are just starting to develop. There is a large standard error (Fig. 5f) and there is no significant effect of ABA+GA at this time point.

Of particular interest in relation to the ABA+GA response was the expression of *PICKLE* a negative regulator of SE which was decreased at week 2 and 4 (Fig. 5g) and the increased expression of *GA2ox* at all time points (Fig. 5h).

## Discussion

### The Utility of the GA+ABA Synergism

GA+ABA usually act antagonistically, with GA frequently stimulating a process and ABA inhibiting [25], [26]. In the case of SE the antagonism is the converse, in general ABA being a positive regulator of SE and GA being a negative regulator of SE [24]. To an extent this latter GA:ABA antagonism is evident in *M. truncatula* at high concentrations of GA where it is inhibitory (Fig. 1) and ABA where there is a small stimulation (Fig. 2). However, surprisingly, in the *M. truncatula* system when ABA and GA are applied together from the beginning of culture, GA by interacting with ABA, greatly enhances SE in a synergistic fashion. This is discussed below in the context of gene expression.

While stable transformation of *M. truncatula* 2HA based on SE systems has been available for a long time [30], the efficiency could still be improved. In particular there is an increasing need for high throughput transformation in plant biology, so it was important to check that the addition of GA and ABA carried through in *M. truncatula* transformation systems, and this was the case. We have found the new medium more robust in subsequent transformation work, as well as embryogenesis occurring more quickly and in total numbers of embryos formed (Fig. 2, Fig. 3).

Transformation of a number of legume species is possible and transgenic soybean is well established. However the routine genetic transformation in the generally recalcitrant legumes remains difficult [33]. The findings of GA+ABA synergism in the model legume *M. truncatula* could prove useful in improving transformation, in at least some legume species.

### Gene Expression and Implications for the GA+ABA Response

Previous studies in *M. truncatula* on *MtSERK1, MtWUS* and *MtSERF1* have pointed to important roles in SE [19]. It was possible that GA+ABA increased expression of these genes to enhance SE but there is no substantive evidence for this. *MtSERK1* expression is associated with developmental change such that it increases expression in callus formation and in early embryo development [34]. In Arabidopsis overexpression of *SERK1* increases SE [22]. The data here show that GA+ABA cause a slight alteration in the expression pattern but there is no increase in expression. There is evidence that *WUS* expression is associated with the formation of totipotent stem cells in both *M. truncatula* [21] and Arabidopsis [18]. Importantly, *MtWUS* is cytokinin-induced while *WUS* is auxin-induced in Arabidopsis. In Arabidopsis it has been shown that *WUS* acts as a transcriptional repressor to induce SE in roots [35]. The increased early expression at one week could represent an enhancement of this early phase by GA+ABA. Apart from *GA2ox*, *MtWUS* is the only case of increased expression at one week in ABA+GA treatments. A requirement for *MtSERF1* for SE has been shown for *M. truncatula* [13] and for soybean and Arabidopsis [12]. In *M. truncatula* there is a wave of *MtSERF1* expression in the embryogenic 2HA peaking after 2–3 weeks of culture, and there is low expression after 4 weeks [13]. *MtSERF1* is also expressed in zygotic embryogenesis [13]. However ABA+GA do not enhance the expression of this ethylene-responsive gene. Expression of the *MtSERF1* gene in the presence of ABA+GA at week 4 is quite low, reflecting the end of a transcriptional wave as the embryo morphology develops. *MtSERF1* expression becomes confined to the shoot apical region [13], and transcription factors such as *LEC1* start to be expressed (Fig. 5f) for embryo maturation and seed filling.

The *MtSK1* stress kinase gene is induced by wounding the tissue [7] and the further addition of ABA+GA does not change *MtSK1* expression (Fig. 5d). However the *MtRBOHA* expression is reduced by ABA+GA suggesting that there is modulation of ROS production. While ROS may have a signaling role in SE in relation to stress [36], [37] excessive ROS can lead to cell death [38]. The results here could reflect a decreased stress response and shorter recovery consistent with the faster and more efficient SE response. This may well be a contributing factor, but given the literature on GA and ABA [16], [24], [25], [39] and the data here on *PKL* and *GA2ox*, the GA and ABA interaction point to other important regulatory areas.

Investigations of mechanisms of Arabidopsis SE have indicated an important role for reducing GA levels [15], [16], [24], [39]. The GA data we obtained seems incompatible with the Arabidopsis studies given that GA represses the embryonic state [39]. However there are commonalities in that *GA2ox* is stimulated and *PKL* expression is reduced in weeks 2 and 4. *GA2ox* was also up-regulated in microarray studies of *M. truncatula* embryogenic cultures induced from auxin + cytokinin treated protoplasts [13]. *GA2ox* was first isolated in the legume *Phaseolus coccineus* (runner bean) and bioactive GA levels can be reduced by the action of this gene resulting in a range of dwarf phenotypes when overexpressed in Arabidopsis and wheat [40]. GA2-oxidases are involved in a major GA inactivation pathway [41]. In the case of *M. truncatula GA2ox* is possibly stimulated to inactivate some of the bioactive GA but still producing suitable ABA:GA ratios for the SE response, or alternatively having additional roles in *Medicago.* Over expression of *LEC* transcription factors can induce SE in the Arabidopsis situation [42] possibly in part because of its capacity to repress GA levels [24] and influence ABA:GA ratios. However, there is no *LEC1* expression in the *M. truncatula* SE induction phase. Clearly future studies need to analyse actual intracellular bioactive GA and ABA levels.

Henderson et al. [39] have proposed that *PKL*, a repressor of the embryonic state, must be down-regulated to facilitate SE induction [16]. *PKL* is a chromatin remodeling factor that promotes histone methylation to repress transcription [43], [44]. Why then are high exogenous ABA:GA ratios required for SE in Arabidopsis [24] but low ABA:GA ratios in *M. truncatula*? One possibility is that the *PKL* gene must be repressed in both Arabidopsis and *M. truncatula* but it is regulated by different ABA:GA intracellular ratios. This would allow derepression of the embryogenesis genes by chromatin remodeling [44] in both cases. Manipulating PKL levels in Medicago would help resolve the SE relationship.

The current study with *M. truncatula*, taken together with previous investigations, indicates that auxin or auxin plus cytokinin dependent SE requires appropriate levels of other endogenous hormones. In *M. truncatula* [13], Arabidopsis and soybean [12], [45] suitable levels of ethylene as well as suitable GA:ABA ratios appear necessary [14], [24]. In different species or cultivars the same gene may be regulated by different hormones or different hormone ratios in regulating SE. In Arabidopsis SE auxin induces *WUS* [18] while it is cytokinin in *M. truncatula* [21]; in Arabidopsis down-regulation of *PKL* expression is linked to high ABA:GA ratios [39] and low ABA:GA ratios in *M. truncatula*. It is reasonable to assume that gene networks provide the co-ordination that is characteristic of the species. With extensive experimentation of SE in a number of systems including Arabidopsis [12], [46], [47], Medicago [19], Brassica [48] and Norway spruce [49] as well as high throughput studies in a range of species such as potato [50] and the rubber tree [51]; it should be possible to develop a better understanding of the way different gene networks can regulate SE in the species of interest.

## Conclusions

The ABA and GA synergism in enhancing somatic embryogenesis in *M. truncatula* has implications for facilitating transformation and in understanding the mechanism of SE. Stable transformation in *M. truncatula* (as opposed to transgenic hairy roots) is still not readily utilised in this model legume and enhanced regeneration is very helpful in this regard. The *M. truncatula* findings may well be useful for transformation of other legumes. While more detailed analysis of the *PICKLE* gene (a likely repressor of the embryonic state) is required, it is of particular interest that this gene is down-regulated by using low ABA:GA ratios in *M. truncatula* whereas high ABA:GA ratios are required in Arabidopsis. Different species may require a different hormone complement in order to regulate the same key genes central to SE in higher plants.

## Materials and Methods

### Plant Materials

*M. truncatula* 2HA plants were glasshouse grown with night/day temperatures of 19/23°C and day length of 14 h.

### Tissue Culture

The details of culturing 2HA leaves for producing somatic embryos were as described by Nolan and Rose [10], [52]. 2HA leaves were sterilised and explants cut as described [52] and plated abaxial side down on the culture plate. The standard culture media is P4 10∶4 (NAA:BAP in µM) for the first 3 wks and P4 10∶4∶1 (NAA:BAP:ABA in µM) for the remainder of culture with sub-culturing every 3–4 weeks [10]. GA was added to the experimental medium at concentrations indicated. The GA+ABA gene expression experiments used P4 10∶4∶1∶5 (NAA:BAP:ABA:GA in µM) for the whole culture period with the control P4 10∶4 (NAA:BAP in µM). Sub-culturing was performed every 3–4 weeks.

### Sample Collection for Gene Expression Studies

The calli in culture plates were harvested at 1, 2 and 4 weeks. The tissue was snap-frozen in liquid nitrogen and kept in a −80°C freezer for later use.

### Plant Transformation Tests

The method of *Medicago* transformation was as described by Nolan et al. [34] and Song et al. [27]. In the transformation, there are three phases i) coculturing leaf explants and the AGL1 *Agrobacterium* strain using P4 10∶4 medium for 2 days ii) culturing using P4 10∶4 plus timentin and selection antibiotic hygromycin for 3 weeks iii) culturing using P4 10∶4∶1 plus timentin and hygromycin with sub culturing every 3–4 weeks. In the ABA+GA treatment, we used P4 10∶4∶1∶5 to replace P4 10∶4 and P4 10∶4∶1 and kept the same antibiotics (see Table 1). A construct of *MtOLEOSIN4* GFP (Ole4-GFP) and its control GFP (Null-GFP) in the Gateway compatible binary vector pMDC83 was used for transformation tests.

### Expression of Somatic Embryogenesis Related Genes

The time points selected represented leaf explants undergoing dedifferentication (1 week), callus formation (2 week) and transition to somatic embryo emergence (4 week) based on our previous studies [7], [19]. To maintain consistency with 1 week and 2 weeks, we did not add ABA at 3 weeks to the auxin and cytokinin controls in the gene expression studies, focusing on the large ABA+GA effect (when added to auxin and cytokinin) relative to auxin and cytokinin alone.

RNA was isolated from sampled calli using the RNAqueous-4PCR kit (Ambion) and DNase treated according to the manufacturer’s instructions. Synthesis of cDNA was performed with a SuperScript III first-strand synthesis system (Invitrogen) using 2 µg of total RNA and oligo (dT) primers. The cDNA was diluted 1∶25 for quantitative PCR (qRT-PCR) reactions. All qRT-PCR reactions were prepared using a CAS1200 robot (Qiagen) and run on a Rotor-Gene Q (Qiagen). Primers (Table B in File S1) were designed using Primer3 and used to amplify specific genes. Information on the individual genes [7], [13], [21], [23], [53], [54] can be found in Tables A and C in File S1. Reactions were performed in duplicate (15 µL sample volume) using Platinum Taq PCR polymerase, 2 µM SYTO9 fluorescent dye (Invitrogen), primers at 0.4 µm and 0.2 mM dNTPs. PCR cycling conditions were 94°C for 2 min, followed by 40 cycles of 94°C for 15 s, 60°C for 30 s and 72°C for 30 s. Disassociation analysis was performed for each run to verify the amplification of a specific product. The *GAPDH* gene was used as a calibrator. *GAPDH* is a suitable reference gene for *M. truncatula* based on geNORM software [55] and our previous microarray and qRT-PCR studies on SE [13]. Three biological repeats were carried out with duplicate reactions. PCR efficiency of each run was calculated using the LinRegPCR program [56]. Relative expression was calculated using the Pfaffl method [57]. Expression of all genes except *MtLEC1* was calibrated to expression in explant source leaf tissue (given the relative expression of 1). *MtLEC1* expression was calibrated to expression in P4 10∶4∶1∶5 medium at 4 weeks as *MtLEC1* has no detectable expression in leaf tissue. Results shown are means ± SE of three biological repeats.

### Statistical Analysis

The statistical analysis on the comparison between multiple treatments was performed by comparing means in JMP10.0 (SAS Institute, Cary, NC).

## Supporting Information

File S1 **Table A:** Medicago gene name and locus. **Table B:** qRT-PCR primer sequences. **Table C:** Medicago gene loci and Arabidopsis homologues.
(DOCX)
