# Supplementary material for: An Unusual Abscisic Acid and Gibberellic Acid Synergism Increases Somatic Embryogenesis, Facilitates Its Genetic Analysis and Improves Transformation in Medicago truncatula
Source: PLoS One. 2014 Jun 17;9(6):e99908. doi: 10.1371/journal.pone.0099908 (PMC4061021; doi:10.1371/journal.pone.0099908)
Supplement: File S1 — Table A: Medicago gene name and locus. Table B: qRT-PCR primer sequences. Table C: Medicago gene loci and Arabidopsis homologues. (DOCX) [file pone.0099908.s001.docx]

**Supplementary File S1**

**Table A:** Medicago gene name and locus

| **Gene name** | **Reference Number*** | **Medicago Locus^1^** |
| --- | --- | --- |
| *MtSERK1* | (23) Nolan et al. 2003 | GenBank: AY162177.1 |
| *MtWUSCHEL* | (21) Chen et al. 2009 | Medtr5g021930 |
| *MtSERF1* | (13) Mantiri et al. 2008 | Medtr1g040430 |
| *MtSK1* | (7) Nolan et al. 2006 | Medtr2g006330 |
| *MtLEC1* | (53) Song 2013 | Medtr1g039040 |
| *MtRBOHA* | (54) Marino et al.2011 | Medtr8g095520 |
| *MtPICKLE* | Table C | Medtr3g106210 |
| *MtGA2ox1* | Table C | DFCI: TC180374 |

** M. truncatula* specific gene publication

DFCI (Dana Farber Cancer Institute) Gene Index Project <http://compbio.dfci.harvard.edu/tgi/plant.html>

^1^Obtained from the Mt3.5 genome release (http://www.medicagohapmap.org).

**Table B:** qRT-PCR primer sequences

| **Gene name** | **Forward (5’-3’)** | **Reverse (5’-3’)** |
| --- | --- | --- |
| *MtSERK1* | CCAAGTGACCTGGGGAATCTTAC | GCTGTTGTTATTAAGCCGCAGAA |
| *MtWUSCHEL* | CTTACAACATTTCATCTGCTGGGCT | CGACATGATGACCAATCCATCCTAT |
| *MtSERF1* | TCATACGCCATCATCTCTTAGGT | AGGGGTTGTTTCCTTTGAAGAT |
| *MtSK1* | AAGACGAGGCTCGATATTTCTTCC | CCAATAAGGTGTTCTCGAGCTTCA |
| *MtLEC1* | ATGGAAAAGAAGGAGGCAAAGC | CAATGGGGAAACTTGGTTTTGA |
| *MtRBOHA* | Gatgtatctggctattcccatcacc | tttccaggataaacagccaccttc |
| *MtPICKLE* | GGGCTGAAAGACAAATACGGATAG | ATGAGGATTCCAATCACTGTCATA |
| *MtGA2ox1* | AGCAAGCCTGTGATGGATAAACA | TCTGTGTGTTCTCCAAACCCAAT |

**Table C:** Medicago gene loci and Arabidopsis homologues

| **Gene name** | **Medicago locus**^1^ | **Arabidopsis locus** | **Gene name** | **Amino acid identity** |
| --- | --- | --- | --- | --- |
| *MtPICKLE* | Medtr3g106210 | AT2G25170 | *PICKLE* | 62.1% |
| *MtGA2ox1* | TC180374 | AT1G78440 | *GA2ox1* | 55.4% |

^1^Obtained from the Mt3.5 genome release (http://www.medicagohapmap.org).
